# Renal failure after surgical mitral valve interventions: a meta-analytical approach

**DOI:** 10.1097/MS9.0000000000002422

**Published:** 2024-08-14

**Authors:** Hasaan Ahmed, Mahmoud Ismayl, Anirudh Palicherla, Ruth Ann Mathew Kalathil, Jalal Dufani, Amjad Kabach, Ahmed Aboeata

**Affiliations:** a Department of Medicine, Division of Internal Medicine, Creighton University School of Medicine, Omaha, NE; b Department of Cardiovascular Medicine, Mayo Clinic, Rochester, MN; c Department of Medicine, Division of Cardiovascular Disease, Creighton University School of Medicine, Omaha, NE, USA

## Introduction

Mitral valve disease remains a significant burden on the global population, propagated by its complex anatomy and wide spectrum of inciting causes^1^. Individuals afflicted with mitral valve disease are at a high risk of deterioration, with heart failure and sudden cardiac death being feared complications. While an array of procedural modalities exist in treating mitral valve disease, surgical intervention remains the gold standard, encompassing either mitral valve repair (MVr) or replacement (MVR).

Renal failure is a feared complication among those undergoing cardiac surgery, associated with profound morbidity and mortality, with data on the incidence of renal failure after surgical mitral valve interventions remaining limited^2^. Therefore, we conducted a meta-analysis of several studies to compare outcomes of renal failure following surgical mitral valve repair versus replacement.

## Methods

We performed a meta-analysis to evaluate the outcomes of renal failure after surgical mitral valve interventions. A comprehensive literature search was performed for studies evaluating outcomes of renal failure in patients undergoing surgical MVr vs. MVR. We searched PubMed, EMBASE, ClinicalTrials.gov, and Cochrane databases with the following search terms: ‘mitral valve surgery’, ‘renal failure’, ‘surgical mitral valve repair’, and ‘surgical mitral valve replacement’. We excluded systematic reviews, meta-analyses, case reports, case series, and cross-sectional as they lacked a control group for comparison. A two-step screening process was used by two evaluators for title, abstract, and full-text screening. Any disagreements that arose among reviewers were addressed through extensive discussions. A random-effects model was used to calculate risk ratios (RRs) with a 95% CI for renal failure. Heterogeneity was assessed using the *I* ^2^ test.

## Results

**Figure 1:**
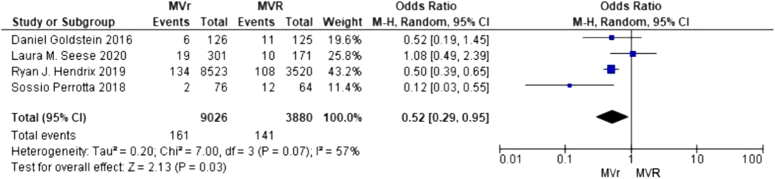
Renal failure after surgical mitral valve interventions.

A total of four studies were identified consisting of one randomized controlled trial and three retrospective observational studies^3–6^. The total study population consisted of 12 906 patients of which 9026 underwent MVr and 3880 underwent MVR. Compared to MVR, MVr was associated with a significantly decreased risk of renal failure postoperatively (RR 0.52; 95% CI: .29–.95; *P*=0.03 (Fig. 1).

## Discussion

The decreased risk of renal failure among patients who underwent MVr compared to MVR is congruent with prior studies evaluating clinical outcomes of mitral valve surgery. In a retrospective study by Kurlansky *et al*.^7^, elderly patients who underwent MVr had a significantly longer median survival time (11.3 years) than those who underwent MVR (6.9 years) (*P*<0.001). Similarly, Gorav *et al*.^8^ found mortality to be significantly higher in those who underwent MVR (23.4%) compared to MVr (7.1%) (*P*=0.01). Interestingly, when stratified by age, postoperative kidney failure was found to be similar between patients <75 years old who underwent MVr compared to MVR (*P*=0.17), however, acute kidney failure was more frequent among those ≥75 years old who underwent MVR compared to those who underwent MVr (*P*=0.03)^8^. Given that our results are age-dependent, further studies should be conducted to compare the incidence of renal failure following surgical mitral valve interventions, stratified by age.

Our findings can be explained by conservation of the subvalvular apparatus during MVr, resulting in decreased mortality and enhanced left ventricular function^8^. Decreased duration of cardiopulmonary bypass during MVr likely also explains our findings, with prior studies noting increasing cardiopulmonary bypass time to be significantly associated with acute renal failure^8^.

There remains a significant debate regarding the appropriate surgical intervention for mitral valve disease^9^. While prior studies have noted decreased operative risks of morbidity and mortality with MVr, attributed to timely intervention and enhanced risk stratification, other studies have found MVR to be superior in reducing the reoccurrence of mitral valve regurgitation and rehospitalizations after surgical intervention^9^. The ongoing uncertainty surrounding the superiority of either surgical intervention is further compounded by the absence of randomized controlled trials comparing outcomes between MVr and MVR, along with previous studies failing to demonstrate significant evidence favoring one surgical approach over the other^9–11^.

## Conclusion

Patients with mitral valve disease are at an increased risk for renal failure, driven by their multiple comorbidities, with this risk further amplified in the setting of cardiac surgery^2^. This meta-analysis found surgical MVr to be associated with a decreased risk of renal failure compared to surgical MVR, with our findings suggesting that MVr should be considered superior to MVR in surgical practice. This study may serve as a framework for the consideration of surgical interventions in patients with mitral valve abnormalities. Further studies are warranted to confirm our findings.

## Ethical approval

Not applicable.

## Consent

Not applicable.

## Source of funding

Not applicable.

## Author contribution

H.A., M.I., A.P., and R.K.: conceptualization; H.A., M.I., A.P., and R.K.: writing – original draft; H.A., M.I., A.P., R.K., J.D., A.K., and A.A.: writing – review and editing; H.A., M.I., A.P., and R.K.: investigation; J.D., A.K., and A.A.: supervision.

## Conflicts of interest disclosure

All authors have reported that they have no conflicts of interest or potential conflicts of interest concerning the contents of this manuscript.

## Research registration unique identifying number (UIN)

Not applicable.

## Guarantor

Hasaan Ahmed MD.

## Data availability statement

The data which supports the findings of this manuscript are openly available upon reasonable request from the corresponding author.

## Provenance and peer review

Not commissioned, externally peer-reviewed.
